# Arrow of time and its reversal on the IBM quantum computer

**DOI:** 10.1038/s41598-019-40765-6

**Published:** 2019-03-13

**Authors:** G. B. Lesovik, I. A. Sadovskyy, M. V. Suslov, A. V. Lebedev, V. M. Vinokur

**Affiliations:** 1 Moscow Institute of Physics and Technology, Institutskii per. 9, Dolgoprudny, 141700 Moscow District, Russia; 2 Materials Science Division, Argonne National Laboratory, 9700S. Cass Avenue, Argonne, Illinois 60637 USA; 3 Theoretische Physik, ETH Zürich, Wolfgang-Pauli-Strasse 27, CH-8093 Zürich, Switzerland

## Abstract

Uncovering the origin of the “arrow of time” remains a fundamental scientific challenge. Within the framework of statistical physics, this problem was inextricably associated with the Second Law of Thermodynamics, which declares that entropy growth proceeds from the system’s entanglement with the environment. This poses a question of whether it is possible to develop protocols for circumventing the irreversibility of time and if so to practically implement these protocols. Here we show that, while in nature the complex conjugation needed for time reversal may appear exponentially improbable, one can design a quantum algorithm that includes complex conjugation and thus reverses a given quantum state. Using this algorithm on an IBM quantum computer enables us to experimentally demonstrate a backward time dynamics for an electron scattered on a two-level impurity.

## Introduction

A fundamental question of the origin of irreversibility of time emerged already in classical statistical physics^1–5^ and has been remaining ever since a subject of an continuous attention^6–8^. Intense researches revealed several aspects of this problem. One of them is a statistical mechanics view discussing the irreversibility problem in the context of the fluctuation theorem^9–16^. In particular, it was quantitatively described and shown experimentally that in a finite temporal interval the time reversed dynamics can emerge^17^. The quantum systems were discussed in^18^ where the positive entropy production rate was experimentally demonstrated on a single spin-1/2 particle, while in^19^ the negative entropy production rate in the presence of a Maxwell’s Demon was observed for spin-1/2 quantum system. Moreover, the full quantum treatment have shown theoretically^20,21^ and later experimentally^22^ that the presence of initial mutual correlations between subparts of a quantum system may lead to a local violation of thermodynamical laws and hence to the thermodynamic arrow of time reversal. Even in a quantum system initially not correlated with an environment, the local violation of the Second Law can occur, as it was demonstrated, with the mathematical rigor^23^, in the framework of the quantum channel theory^24^. Most of the above works were based in a good part on thermodynamic considerations. From the slightly different perspective this question was discussed in the seminal work by Zurek^25^, who looked at the irreversibility issue from the angle of the loss of predictability with the time. A solely quantum mechanical aspect of the problem was stressed by Landau^26^ and von Neumann^27^ who related irreversiblity to the process of a macroscopic measurement. In^28^ the arrow of time dilemma was addressed from the point of view system-observer considerations, but later this approach was criticized in^29^. Here, in the spirit of quantum mechanics, we elaborate on the implications of the Wigner’s result^30^ that time reversal operation is anti-unitary because it requires complex conjugation. We demonstrate that this emerging anti-unitarity predicates that the universal time reversal operation does not spontaneously appear in nature. To make the time reversal possible, one would need a supersystem manipulating the quantum system in question. In most of the cases, such a supersystem cannot materialize spontaneously. As an illustration, we use the simplest systems of a single- or two particles subject to electromagnetic fluctuations. We show that even the evolution of these single- or two-particle states in a free space generates the complexity that renders spontaneous time reversal either highly improbable or actually impossible. We expect that if irreversibility emerges even in the systems that simple, than, even, more it should appear in the more complex systems. In what follows, we quantify the complexity of the preparation of the time-reversed quantum state and the probability of its spontaneous emergence. We show that the time-reversal complexity of the developed quantum state scales linearly with the dimension of the Hilbert space swept by the system in the course its forward time evolution, but that one can devise an administering supersystem artificially. This is implemented experimentally by modeling a real system, the electron scattered on the two-level systems, on the IBM quantum computer. In this respect we utilize the conjectures by Lloyd^6^.

Further, a principal possibility of occurring of the time reversal was discussed in^20^.

## Reversal of The Spreading Wave Packet

**Figure 1:**
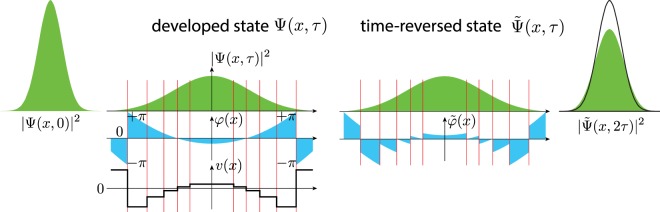
Time reversal procedure for a Gaussian wave-packet ${\rm{\Psi }}(x,0)\propto \exp (\,-\,{x}^{2}/2{\sigma }^{2})$, $\sigma =1(a.u.)$. The wave-packet spreads ${\rm{\Psi }}(x,0)\to {\rm{\Psi }}(x,\tau )$ according to a quadratic Hamiltonian ${\hat{p}}^{2}/2m$ during the time interval $\tau =3m{\sigma }^{2}/\hslash$. At the moment $\tau$ the system is exposed to the fast step-wise electromagnetic potential fluctuation *v*(*x*) (second panel). The fluctuation approximately (with the precision corresponding to the density of partitioning points) conjugates the phase of the wave-function: $\phi (x,{\tau }^{-0})\to \tilde{\phi }(x,{\tau }^{+0})=\phi (x,{\tau }^{-0})+ev(x,\tau )\delta \tau /\hslash$ (third panel). The prepared time-reversed state $\tilde{{\rm{\Psi }}}(x,\tau )$ then freely evolves during the same time interval $\tau$ and arrives to the squeezed state $\tilde{{\rm{\Psi }}}(x,2\tau )$ (fourth panel). The resulting state $\tilde{{\rm{\Psi }}}(x,2\tau )$ has 86% overlap with the initial state ${\rm{\Psi }}(x,0)$ shown as an empty envelope curve in the fourth panel.

That in quantum mechanics in order to execute a time reversal operation one has to perform complex conjugation of the wave function, implies that the time reversal operator $\hat{{\mathscr{T}}}$ is a product of a complex conjugation operator $\hat{{\mathscr{K}}}$ and a unitary rotation ${\hat{U}}_{R}$, i.e. $\hat{{\mathscr{T}}}={\hat{U}}_{R}\,\hat{{\mathscr{K}}}$, where for any ${\rm{\Psi }}$, $\hat{{\mathscr{K}}}{\rm{\Psi }}={{\rm{\Psi }}}^{\ast }$. This operation not only reflects velocities like in the classical physics, but also reverses phases of the wave function components. A general universal operation that can reverse any arbitrary wave function, does not exist in nature. Yet, some special ${\rm{\Psi }}$-dependent operation such that ${\hat{U}}_{{\rm{\Psi }}}{\rm{\Psi }}={{\rm{\Psi }}}^{\ast }$ can exist and below we explicitly construct such an operation for a system of qubits. To that end, one has to design a supersystem that is external with respect to the system of interest and which is capable to implement the purposeful manipulating on the given system. In nature, in the simplest case of a single particle, the role of such a supersystem can be taken up, for example, by the fluctuating electromagnetic field. To gain an insight into how this works, let us consider a wave packet corresponding to the particle with the square energy dispersion, $\varepsilon ={p}^{2}/2m$, where *p* is the particle momentum and *m* is the particle mass, propagating in space, see Fig. 1. The electromagnetic field is assumed to be predominantly weak except for rare fluctuations. Thus, the spreading of the wave packet is coherent. At large times $\tau$ the wave packet spreads as

1 $${\rm{\Psi }}(x,\tau )\simeq \frac{f(xm/\hslash \tau )}{\sqrt{2\pi \hslash \tau /m}}\,\exp \,(i\frac{m{x}^{2}}{2\hslash \tau }),$$

where *f*(*q*) is a Fourier image of the initial spatial wave function. The phase of ${\rm{\Psi }}$ changes as a result of the action of the fast fluctuation of an external potential, i.e. with the potential that changes on the times much shorter than the characteristic time of the phase change. To set the fluctuation that complex conjugates ${\rm{\Psi }}$, let us divide the coordinate space into a large number of the elemental cells *δx*_*n*_ where a wavefunction’s phase $\phi (x,\tau )$ changes slowly and look for a fast electromagnetic potential fluctuation $V(x,t)$ which is smooth on the cell’s scale and reverts the phase of the wavefunction: $\int \,dt\,eV({x}_{n},t)/\hslash =-\,2\phi ({x}_{n},\tau )$. If during the $\tau$ the wave packet (1) has spread from the size *L*_0_ to the size ${L}_{\tau }=\hslash \tau /m{L}_{0}$, it would require $N\sim {\epsilon }^{-1/2}({L}_{\tau }/{L}_{0})$ elementary cells to approximately revert the quantum state ${\rm{\Psi }}(x,\tau )\to {\tilde{{\rm{\Psi }}}}^{\ast }(x,\tau )$ with the probability $1-\epsilon$: $|\langle {\tilde{{\rm{\Psi }}}}^{\ast }(x,\tau )|{{\rm{\Psi }}}^{\ast }(x,\tau )\rangle {|}^{2}=1-\epsilon$, see Supplementary Information (SI). Then the probability of the spontaneous reversal, i.e. the probability of the appearance of the required electromagnetic potential fluctuation, estimates as 2^−*N*^. Now we determine the typical time scale $\tau$ on which the spontaneous time reversal of a wave-packet can still occur within the universe lifetime ${t}_{U}\sim 4.3\times {10}^{17}$ sec. The latter is obtained from the estimate ${2}^{-N}\simeq \tau /{t}_{U}$, where the number of cells $N\sim {\epsilon }^{-1/2}\,(\langle E\rangle \tau /\hslash )$ is expressed through the average particle energy $\langle E\rangle ={\hslash }^{2}/m{L}_{0}^{2}$. As a typical average energy of the wave-packet we take the energy corresponding to the current universe temperature 2.72 K, and arrive at $\tau \simeq 6\times {10}^{-11}$ sec. One thus sees that even in the discussed simplest possible example of a single quantum particle the time reversal is already a daunting task where even with the GHz rate of attempts, the required fluctuation is not observable within the universe lifetime. The above arguments reveal that, in quantum mechanics, time irreversibility emerges already on the level of a single evolving particle.

Now we consider a more complex example and demonstrate that a separable state

2 $${\rm{\Psi }}({x}_{1},{x}_{2})=|{\psi }_{1}({x}_{1}){\psi }_{2}({x}_{2})|\,\exp \,[i({\phi }_{1}({x}_{1})+{\phi }_{2}({x}_{2}))]$$

of two particles can not be reverted by classical field fluctuations in the case where particle’s wave functions overlap. Let all particles have the same electric charge *q* and interact with a classical electric potential *v*(*x*, *t*). The potential fluctuations produce phase shifts $\int \,dtqv(x,t)/\hslash$. Accordingly the proper fluctuations capable to reverse the quantum state should satisfy the condition ${\phi }_{1}({x}_{1})+{\phi }_{2}({x}_{2})$ + $\int \,dt[qv({x}_{1},t)+qv({x}_{2},t)]/\hslash$ = $-\,{\phi }_{1}({x}_{1})-{\phi }_{2}({x}_{2})$. For ${x}_{1}={x}_{2}$ it implies $\int \,dtqv(x,t)/\hslash =-\,{\phi }_{1}(x)-{\phi }_{2}(x)$, and therefore at ${x}_{1}\ne {x}_{2}$ one has to satisfy the condition ${\phi }_{2}({x}_{2})+{\phi }_{1}({x}_{1})={\phi }_{2}({x}_{1})+{\phi }_{1}({x}_{2})$ which, in general, does not hold.

Quantum entanglement introduces the next level of complexity for the time-reversal procedure. Consider a two-particle state $${\rm{\Psi }}({x}_{1},{x}_{2})=|{\rm{\Psi }}({x}_{1},{x}_{2})|{e}^{i\phi ({x}_{1},{x}_{2})}$$ with the non-separable phase function $$\phi ({x}_{1},{x}_{2})={a}_{1}({x}_{1}){b}_{1}({x}_{2})+$$$${a}_{2}({x}_{1}){b}_{2}({x}_{2})$$. In this situation even for the non-overlapping particles with $${\rm{\Psi }}({x}_{1},{x}_{2})=0$$ for $${x}_{1}={x}_{2}$$ the two-particle state can not be reversed by an interaction with classical fields. Let one access the particles by different fields which induce separate phase shifts $${\rm{\Psi }}({x}_{1},{x}_{2})\to {\rm{\Psi }}({x}_{1},{x}_{2}){e}^{i({\varphi }_{1}({x}_{1})+{\varphi }_{2}({x}_{2}))}$$. The induced phase shifts should satisfy the relation: $${\varphi }_{1}({x}_{1})+{\varphi }_{2}({x}_{2})=-\,2\phi ({x}_{1},{x}_{2})$$, therefore for any three points $${x}_{1}\ne {x}_{2}\ne {x}_{3}$$ the following conditions should hold

3 $${\varphi }_{1}({x}_{1})+{\varphi }_{2}({x}_{2})=-\,2({a}_{1}({x}_{1}){b}_{1}({x}_{2})+{a}_{2}({x}_{1}){b}_{2}({x}_{2})),$$

4 $${\varphi }_{1}({x}_{1})+{\varphi }_{2}({x}_{3})=-\,2({a}_{1}({x}_{1}){b}_{1}({x}_{3})+{a}_{2}({x}_{1}){b}_{2}({x}_{3})).$$

Subtracting these relations one gets ${\varphi }_{2}({x}_{2})-{\varphi }_{2}({x}_{3})$ = $-\,2{a}_{1}({x}_{1})\,({b}_{1}({x}_{2})-{b}_{1}({x}_{3}))$ − $2{a}_{2}({x}_{1})\,({b}_{2}({x}_{2})-{b}_{2}({x}_{3}))$ where the left hand side does not depend on *x*_1_ and therefore one has to assume *a*_1_ and *a*_2_ to be constant. This, however, contradicts the non-separability assumption for $\phi ({x}_{1},{x}_{2})$.

An entangled two-particle state with a non-separable phase function can naturally emerge as a result of scattering of two localized wave-packets^31^. However, as we have seen, the generation of the time-reversed state, where a particle gets disentangled in the course of its forward time evolution, requires specific two-particle operations which, in general, cannot be reduced to a simple two-particle scattering.

The above consideration enables us to formulate important conjectures about the origin of the arrow of time: (i) *For the time reversal one needs a supersystem manipulating the system in question*. *In the most of the cases*, *such a supersystem cannot spontaneously emerge in nature*. (ii) *Even if such a supersystem would emerge for some specific situation*, *the corresponding spontaneous time reversal typically requires times exceeding the universe lifetime*.

A matter-of-course supersystem of that kind is implemented by the so-called universal quantum computer. It is capable to efficiently simulate unitary dynamics of any physical system endowed with local interactions^32^. A system’s state is encoded into the quantum state of the computer’s qubit register and its evolution is governed by the quantum program, a sequence of the universal quantum gates applied to the qubit register. There exists a panoply of ways by which a quantum state of a system can be encoded into the states of the quantum computer. Indeed, choosing a proper dimension of the quantum computer register one can swap its state $|{\psi }_{0}{\rangle }_{{\rm{reg}}}$ with the system’s quantum state, $|{\rm{\Psi }}{\rangle }_{{\rm{sys}}}$, by the unitary operation ${\hat{U}}_{{\rm{SWAP}}}|{\psi }_{0}{\rangle }_{{\rm{reg}}}\otimes |{\rm{\Psi }}{\rangle }_{{\rm{sys}}}=|\psi {\rangle }_{{\rm{reg}}}\otimes |{{\rm{\Psi }}}_{0}{\rangle }_{{\rm{sys}}}$, where the mapping $|{\rm{\Psi }}{\rangle }_{{\rm{sys}}}\to |\psi {\rangle }_{{\rm{reg}}}$ completes the encoding task. Such an encoding procedure is universal i.e. it does not require the knowledge of the system state $|{\rm{\Psi }}{\rangle }_{{\rm{sys}}}$. However, non-physical encodings might be suggested which can not be accomplished by unitary transformation. One of the ways to do that was proposed in^33^ where the real and the imaginary components of the system’s wave function were separately mapped onto the different Hilbert subspaces of the auxiliary system, i.e. quantum computer. Within this representation of the initial quantum system, the complex conjugation can be formulated as a universal unitary rotation of the wave function of the auxiliary system. However, the mapping itself is not a universal unitary operation as follows from the superposition principle arguments. This means that the approach of^33^ merely lifts the problem of the non-unitarity of the quantum conjugation hiding it in the non-unitarity of the mapping procedure. At variance, in what follows we address the time reversal of the original physical system without nonphysical mapping it on some completely different system unrelated to the original one. We start with formulating general principles of constructing time-reversal algorithms on quantum computers and, in the next section, present a practical implementation of a few-qubit algorithm that enabled experimental time reversal procedure on the public IBM quantum computer.

## General Time Reversal Algorithms

Consider a quantum system initially prepared in the state ${\rm{\Psi }}(t=0)$ and let it evolve during the time $\tau$ into the state ${\rm{\Psi }}(\tau )=\exp (\,-\,iH\tau /\hslash ){\rm{\Psi }}(0)$. Let us find a minimal size of a qubit register needed to simulate the dynamics of a system ${\rm{\Psi }}(0)\to {\rm{\Psi }}(\tau )$ with a given fidelity $1-\epsilon$. Let us choose a finite set of time instances ${t}_{i}\in [0,\tau ]$, $i=0,\ldots N^{\prime}$ subject to a condition $|\langle {\rm{\Psi }}({t}_{i})|{\rm{\Psi }}({t}_{i+1}\rangle ){|}^{2}=1-\epsilon$ with ${t}_{0}=0$ for some small $\epsilon  > 0$. Then at any time instant $t\in [0,\tau ]$ a state ${\rm{\Psi }}(t)$ can be approximated by the discrete set of states $\{{\rm{\Psi }}({t}_{i}),i=0,\ldots {\mathscr{N}}^{\prime} \}$ with the fidelity $1-\epsilon$. The set of states $\{{\rm{\Psi }}({t}_{i})\}$ spans the Hilbert subspace ${\mathscr{S}}$ of the dimension ${\mathscr{N}}\le {\mathscr{N}}^{\prime}$. Therefore, ${\mathscr{N}}$ basis vectors $|{e}_{i}\rangle \in {\mathscr{S}}$ can be represented by ${\mathscr{N}}$ orthogonal states of the qubit register, $|{e}_{i}\rangle \to |{\overrightarrow{b}}_{i}\rangle \equiv |{b}_{0}{b}_{1}\ldots \rangle$. The corresponding qubit Hamiltonian $\hat{H}$ which mimics the original Hamiltonian $\hat{ {\mathcal H} }$ is then defined by the relation ${(\hat{H})}_{ij}\equiv \langle {\overrightarrow{b}}_{i}|\hat{H}|{\overrightarrow{b}}_{j}\rangle =\langle {e}_{i}|\hat{ {\mathcal H} }|{e}_{j}\rangle$.

Below we introduce two encoding procedures $|{e}_{i}\rangle \to |{\overrightarrow{b}}_{i}\rangle$. In the first, *sparse* coding approach, one assigns a separate qubit to each state $|{e}_{i}\rangle$, $i\in [0,{\mathscr{N}}-1]$ and encodes the state $\psi (\tau )$ into the ${\mathscr{N}}$-qubit state $|\psi \rangle ={\sum }_{i=0}^{{\mathscr{N}}-1}\,{\psi }_{i}\,|{0}_{0}\ldots {1}_{i}\ldots {0}_{{\mathscr{N}}-1}\rangle$. The second approach is a *dense* coding scheme where one records the state $\psi (\tau )$ into a state of $n=\,{\rm{int}}\,[{\mathrm{log}}_{2}\,({\mathscr{N}})]+1$ qubits $|\psi \rangle ={\sum }_{i=0}^{{\mathscr{N}}-1}\,{\psi }_{i}\,|i\rangle$, where int[*x*] is the closest upper integer to *x*: $x\le \,{\rm{int}}\,[x]$, $|i\rangle \equiv |{b}_{0}\ldots {b}_{n-1}\rangle$ is a computational basis state corresponding a binary representation of the number $i={\sum }_{k=0}^{n-1}\,{b}_{k}{2}^{n-1-k}$.

A time-reversal operation $\hat{{\mathscr{T}}}$ of the qubit register can be presented as a product $\hat{{\mathscr{T}}}={\hat{U}}_{R}\hat{{\mathscr{K}}}$ of the complex conjugation operator $\hat{{\mathscr{K}}}$, $\hat{{\mathscr{K}}}({\psi }_{i}|i\rangle )\equiv {\psi }_{i}^{\ast }|i\rangle$, and some unitary operator ${\hat{U}}_{R}$, whose form is defined by the Hamiltonian $\hat{H}$, ${\hat{U}}_{R}={\hat{U}}_{H}^{\dagger }{\hat{U}}_{H}^{\ast }$, where $\hat{H}={\hat{U}}_{H}^{\dagger }\,{\rm{diag}}\,\{{E}_{1}\ldots {E}_{n}\}{\hat{U}}_{H}$ see SI. Therefore, ?in order to implement the time-reversal operation $\hat{{\mathscr{T}}}$ one needs to know the Hamiltonian $\hat{H}$ explicitly. Note, that quantum computer is able to simulate unitary dynamics governed by an arbitrary Hamiltonian including those that do not correspond any physical system (for example, some non-local Hamiltonian). It is known, that the joint transformation of the charge conjugation, parity inversion, and time reversal is considered as an exact symmetry of all known laws of physics, and, therefore, the qubit Hamiltonian $\hat{H}$, which corresponds to a real physical system, has to honor this symmetry as well. Therefore, the unitary operation describing evolution of the physical system ${\hat{U}}_{R}$ is generally known and represents a transformation which is inherited from the time-reversal symmetry of the original Hamiltonian $\hat{ {\mathcal H} }$. In particular, if the qubit Hamiltonian $\hat{ {\mathcal H} }$ is real, then the corresponding evolution operator $\hat{U}(\tau )$ is symmetric that entails ${\hat{U}}_{R}={\bf{1}}$.

**Figure 2:**
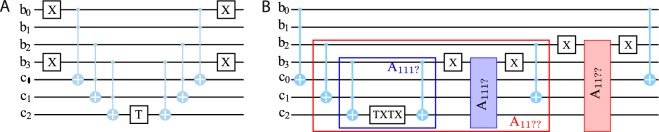
Complex conjugation circuits. (**A**) Quantum circuit implementation of the conditional phase shift operation ${\hat{{\rm{\Phi }}}}_{k=6}$ for a component $\mathrm{|0110}\rangle$. The circuit involves three types of gates: 1-qubit NOT gate $\hat{X}|b\rangle =|b\oplus 1\rangle$, 1-qubit unitary rotation $\hat{T}(\,-\,2{\phi }_{k})[|0\rangle +a|1\rangle ]=|0\rangle +a{e}^{-2i{\phi }_{k}}|1\rangle$, and 3-qubit Toffoli gate which reverts the state of the last target qubit if and only if two first control qubits are both set to $\mathrm{|1}\rangle$: ${\hat{{\rm{\Lambda }}}}_{2}|11\rangle \otimes |b\rangle =|11\rangle \otimes |b\oplus 1\rangle$. The first three Toffoli gates set the ancillary qubit *c*_2_ into $\mathrm{|1}\rangle$ if and only if the qubit register is set to the $\mathrm{|0110}\rangle$ state and the last three Toffoli gates restore the original state $|{b}_{0}{b}_{1}{b}_{2}{b}_{3}\rangle \otimes |000\rangle$. (**B**) The quantum circuit with the optimal Toffoli gate arrangement which conjugates four components: $|1111\rangle$, $\mathrm{|1110}\rangle$, $\mathrm{|1101}\rangle$ and $\mathrm{|1100}\rangle$. The circuit is partitioned into several nested blocks (subroutines) ${A}_{11??}\supset {A}_{111?}$, the question marks standing for an unknown bit value. The first-level block (blue) *A*_111?_ conjugates only computational states where three senior qubits $|{b}_{0}{b}_{1}{b}_{2}\rangle$ are all set to $\mathrm{|1}\rangle$. The next-level block (red) *A*_11??_ contains as a subroutine the block *A*_111?_ and conjugates all components $|{b}_{0}{b}_{1}\rangle =|11\rangle$.

In the following we assume the unitary ${\hat{U}}_{R}$ to be known and focus on the unitary implementation of a complex conjugation operation $\hat{{\mathscr{K}}}$, $\hat{{\mathscr{K}}}\to {\hat{U}}_{\psi }$. In particular, we quantify a complexity of such implementation as a number of elementary quantum gates or/and auxiliary qubits needed to implement ${\hat{U}}_{\psi }$. For a sparse coding scheme, the complex conjugation of the ${\mathscr{N}}$-qubit state $|\psi \rangle ={\sum }_{i=0}^{{\mathscr{N}}-1}\,|{\psi }_{i}|{e}^{i{\phi }_{i}}\,|{0}_{0}\ldots {1}_{i}\ldots {0}_{{\mathscr{N}}-1}\rangle$ can be accomplished by the unitary operation ${\hat{U}}_{\psi }^{(1)}={\prod }_{i=0}^{{\mathscr{N}}-1}\,\otimes {\hat{T}}_{i}(\,-\,2{\phi }_{i})$ where ${\hat{T}}_{i}(\phi )$ is the single qubit operation: ${\hat{T}}_{i}(\phi )|{0}_{i}\rangle =|{0}_{i}\rangle$ and ${\hat{T}}_{i}(\phi )|{1}_{i}\rangle ={e}^{i\phi }|{1}_{i}\rangle$. Consequently, the sparse coding scheme does not require the most “expensive” two-qubit gates at all but do require a large number ${\mathscr{N}}$ of qubits. For the dense coding scheme the situation is the opposite: this scheme involves only a logarithmically smaller number *n* of qubits but instead requires implementation of 2^*n*^ *n*-qubit conditional phase shift operations: $\hat{{\mathscr{K}}}\to {\hat{U}}_{\psi }^{(2)}={\sum }_{j=0}^{{2}^{n}-1}\,|j\rangle \langle j|{e}^{-2i{\phi }_{j}}$ which add proper phases to each component of the state $|\psi \rangle$: ${\hat{U}}_{\psi }^{(2)}|\psi \rangle =|{\psi }^{\ast }\rangle$. Therefore, ${\hat{U}}_{\psi }^{(2)}$ must involve two-qubit gates, i.e. conditional-NOT (CNOT) gates. We quantify the complexity of the dense coding scheme by a number ${N}_{\oplus }$ of CNOT gates needed to implement it. Each phase shift operation ${\hat{{\rm{\Phi }}}}_{i}(\phi )\equiv |i\rangle \langle i|{e}^{i\phi }$ can be build with the help of $n-1$ ancillary qubits and $\mathrm{2(}n-\mathrm{1)}$ Toffoli gates, as shown in Fig. 2A. In total, it requires ${N}_{\oplus }[{\hat{U}}_{\psi }^{(2)}]=12(n-1){2}^{n}\sim 12{\mathscr{N}}\,{\mathrm{log}}_{2}({\mathscr{N}})$ CNOT gates. However, such an arrangement is non-optimal as it involves an excess usage of Toffoli gates. Indeed, let us consider two states, $|j\rangle =|{b}_{0}{b}_{1}\ldots {b}_{n-1}\rangle$ and $|j^{\prime} \rangle =|{b^{\prime} }_{0}{b^{\prime} }_{1}\ldots {b^{\prime} }_{n-1}\rangle$, with coincident two older bits ${b}_{0}={b^{\prime} }_{0}$, ${b}_{1}={b^{\prime} }_{1}$. The separate usage of phase shifts ${\hat{{\rm{\Phi }}}}_{j}({\phi }_{j})$ and ${\hat{{\rm{\Phi }}}}_{j^{\prime} }({\phi }_{j^{\prime} })$ involves the double check of *b*_0_ and *b*_1_ values. The better implementation checks *b*_0_ and *b*_1_ only once and conjugates phases of all states within a set $|{b}_{0}{b}_{1}{b}_{2}\ldots {b}_{n-1}\rangle$ within a separate circuit block. In fact, such optimization can be done for all subsequent junior bits *b*_3_, *b*_4_, see Fig. 2B, that can minimize the usage of Toffoli gates and reduce the reversal complexity to be linear in ${\mathscr{N}}$: ${{\mathscr{N}}}_{\oplus }\sim 24{\mathscr{N}}$, see SI. We thus arrive at the conclusion that *The number of elementary operations needed for the exact time reversal procedure of the dynamics of a quantum system which in the course its evolution sweeps a Hilbert space of a dimension* ${\mathscr{N}}$ *is bounded from above by some number* ${\mathscr{O}}({\mathscr{N}})$. If now we consider typical systems emerging in nature, then the entanglement generates the dimensionality, ${\mathscr{N}}$, that is exponentially large with respect to the number of particles involved.

## Time Reversal Experiment

**Figure 3:**
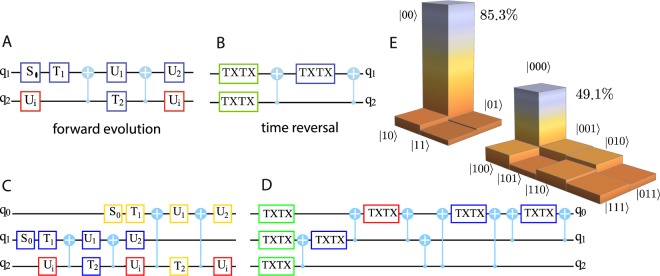
A time-reversal experiment. (**A** and **B**) The quantum circuits which model the scattering process of one or two particles (qubit lines *q*_0_ and *q*_1_) on the two level impurity (qubit line *q*_2_). Unitary operations ${\hat{U}}_{{\rm{i}}}$ (red boxes) describe free evolution of the TLI during the time $\tau$. Remaining operations simulate the particle’s scattering: the group of 1-qubit gates (blue or yellow boxes) combined with the two CNOT gates implements the scattering operator ${\hat{S}}_{\psi }$ for the *q*_1_ or *q*_0_ particle. The parameters of the gates are adjusted in the specific way: ${\hat{T}}_{2}|1\rangle \otimes {\hat{U}}_{2}{\hat{\sigma }}_{x}{\hat{U}}_{1}{\hat{\sigma }}_{x}{\hat{T}}_{1}|q\rangle =|1\rangle \otimes {\hat{S}}_{2}{S}_{1}^{\dagger }|q\rangle$ and ${\hat{T}}_{2}^{\dagger }|1\rangle \otimes {\hat{U}}_{2}{\hat{U}}_{1}{\hat{T}}_{1}|q\rangle =|1\rangle \otimes |q\rangle$, see SI. (**C**) and (**D**) The 2- and 3-qubit quantum circuits realizing the exact complex conjugation procedure. A single qubit gate ${\rm{TXTX}}(\phi ,\bar{\phi })\equiv \hat{T}(\phi ){\hat{\sigma }}_{x}\hat{T}(\bar{\phi }){\hat{\sigma }}_{x}$ performs a phase shift of a qubit components: ${\rm{TXTX}}(\phi ,\bar{\phi })(a{\mathrm{|0}}_{i}\rangle +b{\mathrm{|1}}_{i}\rangle )=a{e}^{i\bar{\phi }}{\mathrm{|0}}_{i}\rangle +b{e}^{i\phi }{\mathrm{|1}}_{i}\rangle$, $i=0,1,3$. The gates TXTX combined properly with the CNOT gates perform the controlled phase shifts associated with the single-qubit (${b}_{i}$, ${\bar{b}}_{i}$, $i=0,1,2$) (green boxes), two-qubit (${b}_{i}\oplus {b}_{j}$, $\overline{{b}_{i}\oplus {b}_{j}}$, $i,j=0,1,2$, $i < j$) (blue boxes) and three-qubit (${b}_{0}\oplus {b}_{1}\oplus {b}_{2}$, $\overline{{b}_{0}\oplus {b}_{1}\oplus {b}_{2}}$) logical term (red box) in the Eq. (5). (**E**) Realization of the 2 or 3-qubit time reversal experiment performed on the IBM public quantum computer. The histogram shows (in percents) the appearance rates of the computational basis states obtained by the 8192 independent runs of the experiment.

Now we are equipped to carry out an experiment implementing two- and three-qubit time-reversal procedures utilizing the public IBM quantum computer. We model a one dimensional particle scattering on a two-level impurity (TLI). The dynamics of the impurity is governed by a Hamiltonian, ${\hat{H}}_{{\rm{i}}}=\hslash \omega (\cos \,\alpha \,{\hat{\sigma }}_{z}+\,\sin \,\alpha \,{\hat{\sigma }}_{x})$. The scattering potential seen by the particle depends on the state of the TLS. The corresponding scattering operator has the form ${\hat{S}}_{i}=|0\rangle \langle 0|\otimes {\hat{S}}_{0}+|1\rangle \langle 1|\otimes {\hat{S}}_{1}$, where ${\hat{S}}_{0}$ and ${\hat{S}}_{1}$ are symmetric unitary scattering matrices of the TLI in a state $\mathrm{|0}\rangle$ or $\mathrm{|1}\rangle$. This scattering problem is modeled by the evolution of the qubit register ${\hat{U}}_{n{\rm{bit}}}|{q}_{i}\rangle \otimes (|{q}_{1}\rangle \otimes \cdots \otimes |{q}_{n}\rangle )$, where $|{q}_{i}\rangle$ qubit describes the state of the TLI and the remaining qubits describe the state of scattered particles. The basis states $|{0}_{i}\rangle$ and $|{1}_{i}\rangle$, $i=1,\ldots n$ correspond to the left and right incoming/outgoing state of the *i*th particle. We consider the processes in which one or two incoming particles are scattered on the freely evolving TLI. We assume that both particles incident from the left being in a well localized ballistically propagating states and arrive at the impurity where they experience instantaneous respective scatterings at separate time instants $t=\tau$ and $t=2\tau$. The corresponding evolutions are described by unitary rotations ${\hat{S}}_{{\rm{i}}}^{(1)}\cdot {\hat{U}}_{{\rm{i}}}(\tau )$ and ${\hat{S}}_{{\rm{i}}}^{\mathrm{(2)}}\cdot {\hat{U}}_{{\rm{i}}}(\tau )\cdot {\hat{S}}_{{\rm{i}}}^{\mathrm{(1)}}\cdot {\hat{U}}_{{\rm{i}}}(\tau )$ for one- and two-particle situation, where ${\hat{U}}_{{\rm{i}}}(\tau )=\exp (\,-\,i{\hat{H}}_{{\rm{i}}}\tau /\hslash )$ describes the free evolution of the TLI between the particles arrivals and ${\hat{S}}_{{\rm{i}}}^{(i)}$, $i=1,2$ is the scattering operator for the *i*-th particle. Next, in both cases we let the system freely evolve during a time $\tau$ that makes the resulting 2-qubit and 3-qubit evolution operators: ${\hat{U}}_{{\rm{2bit}}}={\hat{U}}_{{\rm{i}}}(\tau )\cdot {\hat{S}}_{{\rm{i}}}^{\mathrm{(1)}}\cdot {\hat{U}}_{{\rm{i}}}(\tau )$ and ${\hat{U}}_{{\rm{3bit}}}={\hat{U}}_{{\rm{i}}}(\tau )\cdot {\hat{S}}_{{\rm{i}}}^{\mathrm{(2)}}\cdot {\hat{U}}_{{\rm{i}}}(\tau )\cdot {\hat{S}}_{{\rm{i}}}^{\mathrm{(1)}}\cdot {\hat{U}}_{{\rm{i}}}(\tau )$ more symmetric, that simplifies the form of ${\hat{U}}_{R}$ unitary rotation entering in the time-reversal procedure. The 2-qubit scattering model is endowed with the symmetric evolution operator ${\hat{U}}_{{\rm{2bit}}}$ and, therefore, its time reversal requires only the complex conjugation operation $\hat{{\mathscr{T}}}=\hat{{\mathscr{K}}}$. At variance, the evolution operator ${\hat{U}}_{{\rm{3bit}}}$ of the 3-qubit model is non symmetric and its time reversal requires an additional unitary rotation $\hat{{\mathscr{T}}}={\hat{U}}_{R}\hat{{\mathscr{K}}}$. It follows from the relation ${{\rm{SWAP}}}_{12}\cdot {\hat{U}}_{{\rm{3bit}}}\cdot {{\rm{SWAP}}}_{12}={\hat{U}}_{{\rm{3bit}}}^{t}$, where ${{\rm{SWAP}}}_{12}|{q}_{1}\rangle \otimes |{q}_{2}\rangle =|{q}_{2}\rangle \otimes |{q}_{1}\rangle$ is the swap operation, that the required unitary operation ${\hat{U}}_{R}={{\rm{SWAP}}}_{12}$. The corresponding quantum circuits realizing ${\hat{U}}_{{\rm{2bit}}}$ and ${\hat{U}}_{{\rm{3bit}}}$ are shown on Fig. 3A and C, see the detail in SI.

According to the results of the Section 2, the unitary implementation of the complex conjugation for a 2- or 3-qubit register will require 48 or 144 CNOT gates. These numbers are beyond of the present capability of the IBM public quantum computer due to the finite error rate 1.5–2.5% of its CNOT gates. Here we utilize an alternative to the Section 2 approach (see SI for details), which is based on the arithmetic representation of the *n*-bit AND Boolean function^34^,

5 $$\begin{array}{rcl}{b}_{0}\wedge {b}_{1}\wedge \cdots \wedge {b}_{n-1} & = & \frac{1}{{2}^{n-1}}(\sum _{{i}_{1}}\,{b}_{{i}_{1}}-\sum _{{i}_{1} < {i}_{2}}\,{b}_{{i}_{1}}\oplus {b}_{{i}_{2}}\\  &  & +\,\sum _{{i}_{1} < {i}_{2} < {i}_{3}}\,{b}_{{i}_{1}}\oplus {b}_{{i}_{2}}\oplus {b}_{{i}_{3}}+\cdots +{(-1)}^{n-1}{b}_{0}\oplus \cdots \oplus {b}_{n-1}),\end{array}$$

where ${b}_{0}\wedge {b}_{1}\wedge \ldots$ is 1 if and only if all ${b}_{0}={b}_{1}=\cdots =1$ and ${b}_{0}\oplus {b}_{1}\oplus \ldots$ is modulo 2 addition. Consider, for instance, a 2-qubit situation. The complex conjugation ${\hat{U}}_{\psi }={\sum }_{k=0}^{3}\,{\hat{{\rm{\Phi }}}}_{k}({\phi }_{k})$ can be alternatively represented as

6 $${\hat{U}}_{\psi }=\exp \,[\,-\,2i{\phi }_{00}\,{\bar{b}}_{0}\wedge {\bar{b}}_{1}-2i{\phi }_{01}\,{\bar{b}}_{0}\wedge {b}_{1}-2i{\phi }_{10}\,{b}_{0}\wedge {\bar{b}}_{1}-2i{\phi }_{11}\,{b}_{0}\wedge {b}_{1}],$$

where ${\bar{b}}_{i}$ denotes a negation of the bit value. Summing up all four components in the exponent according to the Eq. (5) one decomposes ${\hat{U}}_{\psi }$ operator into a product of 1-qubit and 2-qubit phase shifts operations:

7 $${\hat{U}}_{\psi }=\exp [\,-\,i{\alpha }_{0}\,{b}_{0}-i{\beta }_{0}\,{\bar{b}}_{0}]\,\exp \,[\,-\,i{\alpha }_{1}\,{b}_{1}-i{\beta }_{1}\,{\bar{b}}_{1}]\,\exp \,[\,-\,i{\alpha }_{01}\,{b}_{0}\oplus {b}_{1}-i{\beta }_{01}\overline{{b}_{0}\oplus {b}_{1}}],$$

where *a*_*i*_, *β*_*j*_ are linear combinations of the phase shifts ${\phi }_{k}$. The modulo 2 addition ${b}_{0}\oplus {b}_{1}$ can be effectively implemented with only two CNOT gates. This approach can be generalized for arbitrary number of qubits and turns out to be more efficient at small *n* since it does not need an ancillary qubits at all and requires $(n-{\mathrm{1)2}}^{n-1}$ CNOT gates for the complex conjugation of an arbitrary *n*-qubit state that wins over the approach discussed in Section 2 for $n\le 48$. In particular, at $n=2$ and 3 one needs only two or eight CNOT gates, respectively. The corresponding 2- and 3-qubit quantum circuits are shown on Fig. 3B and 3D.

The time-reversal experiment runs in several steps: (i) The qubit register that is initially set into the state $|\psi (0)\rangle =|0\ldots 0\rangle$ accomplishes the forward time unitary evolution $|{\psi }_{0}\rangle \to |{\psi }_{1}\rangle ={\hat{U}}_{n{\rm{bit}}}|{\psi }_{0}\rangle$. Next, (ii′) the unitary complex conjugation operation $\hat{{\mathscr{K}}}={\hat{U}}_{\psi }$ is applied $|{\psi }_{1}\rangle \to |{\psi }_{1}^{\ast }\rangle ={\hat{U}}_{\psi }|{\psi }_{1}\rangle$ followed by (ii″) the unitary transformation ${\hat{U}}_{R}$, $|{\psi }_{1}^{\ast }\rangle \to |\hat{{\mathscr{T}}}{\psi }_{1}\rangle ={\hat{U}}_{R}|{\psi }_{1}^{\ast }\rangle$. As a result, the time-reversed state $|\hat{{\mathscr{T}}}{\psi }_{1}\rangle$ is generated. Finally, at step (iii) one applies the same forward time unitary evolution $|\hat{{\mathscr{T}}}{\psi }_{1}\rangle \to {\hat{U}}_{n{\rm{bit}}}|\hat{{\mathscr{T}}}{\psi }_{1}\rangle$ and measures the resulting state of the register in the computational basis. In practice, the step 2″ is only needed for the 3-qubit model where ${\hat{U}}_{R}={{\rm{SWAP}}}_{12}$ requires three additional CNOT gates. In order to save this number of CNOTs we replace the forward evolution operator ${\hat{U}}_{{\rm{3bit}}}$ at step (iii) by the new evolution operation obtained from ${\hat{U}}_{{\rm{3bit}}}$ via the physical interchange of two particle qubits, rather than to implement the SWAP_12_ operation at step (ii″). Generally, to arrive to the same initial state one has to apply the inverse time-reversal operation ${{\mathscr{T}}}^{-1}=\hat{{\mathscr{K}}}{\hat{U}}_{R}^{\dagger }$ to the final state ${\hat{U}}_{n{\rm{bit}}}|\hat{{\mathscr{T}}}{\psi }_{1}\rangle$. However, if the initial state $|\psi \mathrm{(0)}\rangle$ was a product state $|0\ldots 0\rangle$ this operation is in fact not needed. Indeed, the complex conjugation just changes the overall phase of the qubit register while ${\hat{U}}_{R}^{\dagger }$ swaps the same qubit states in 2-particle scattering experiment.

The above time reversal experiment sets the qubit register again into the initial state $\mathrm{|0}\ldots 0\rangle$ with the probability unity, provided all quantum gates are prefect and no decoherence and relaxation processes are present. The exemplary outcome probabilities ${P}_{ij}=|\langle {b}_{i}{b}_{j}|{\tilde{\psi }}_{0}\rangle {|}^{2}$ and ${P}_{ijk}=|\langle {b}_{i}{b}_{j}{b}_{k}|{\tilde{\psi }}_{0}\rangle {|}^{2}$, $i,j,k=0,1$ obtained in a real experiment for the 2- and 3-qubit models are shown on the Fig. 3E. One can see that the probability for observing the correct final state $\mathrm{|0}\ldots 0\rangle$ is less than 100% and for 2- and 3-qubit experiment are given by $85.3\pm 0.4 \%$ and $49.1\pm 0.6 \%$ correspondingly. This considerable distinction from the perfect scenario comes from the three main sources: (i) The finite coherence time *T*_2_ of qubits; (ii) The errors of CNOT gates and (iii) The readout errors of the final state of the qubit register.

The observed outcome probabilities were obtained after 8192 runs of each experiment at the same state of the ‘ibmqx4’ 5-qubit quantum processor, see details in SI. For the 2-qubit experiment two processor’s qubit lines *q*_1_ and *q*_2_ with the coherence times 41.0 *μ*s and 43.5 *μ*s and readout errors $${\epsilon }_{r1}=3.3 \% $$ and $${\epsilon }_{r2}=2.9 \% $$ were involved. For the 3-qubit experiment, the additional *q*_0_ qubit line with $${T}_{2}=39.4\,\mu {\rm{s}}$$ and the readout error $${\epsilon }_{r0}=4.8 \% $$ was used. The 2-qubit experiment requires six CNOT_*q*2,*q*1_ gates with the gate error $${\epsilon }_{g21}=2.786 \% $$, while the 3-qubit experiment acquires, in addition, six $${{\rm{CNOT}}}_{q\mathrm{2,}q0}$$ and four $${{\rm{CNOT}}}_{q\mathrm{1,}q0}$$ gates with the corresponding gate errors $${\epsilon }_{g20}=2.460 \% $$ and $${\epsilon }_{g10}=1.683 \% $$. This numbers give us a rough estimate of the net error rates: $${\epsilon }_{{\rm{2bit}}}=1-{\mathrm{(1}-{\epsilon }_{g21})}^{6}$$$$\mathrm{(1}-{\epsilon }_{r1})\,\mathrm{(1}-{\epsilon }_{r2})\approx \mathrm{15.6 \% }$$ and $${\epsilon }_{{\rm{3bit}}}=1-{\mathrm{(1}-{\epsilon }_{g21})}^{6}{\mathrm{(1}-{\epsilon }_{g20})}^{6}{\mathrm{(1}-{\epsilon }_{g10})}^{4}$$$$(1-{\epsilon }_{r0})(1-{\epsilon }_{r1})(1-{\epsilon }_{r2})$$$$\approx \mathrm{34.4 \% }$$. One can see, that while this estimate agrees with an observed error of a 2-qubit experiment, the error probability for the 3-qubit experiment is underestimated. We argue that a time duration of a single 3-qubit experiment is about 7.5 *μ*s is comparable with *T*_2_ times, while a single 2-qubit experiment takes less time about 3 *μ*s. Hence, the decoherence effects are more prominent in a 3-qubit case that might explain the underestimated value of the error rate. The more experimental data for the different system parameters and processor states are discussed in SI. We note, that at the present date the more accurate computation can be made within NMR quantum computation paradigm^35^, where much more accurate two-qubit gates can be achieved.

## Conclusion

Our findings suggest several directions for investigating time reversal and the backward time flow in real quantum systems. One of the directions to pursue, is the time dependence of the reversal complexity ${\mathscr{N}}$ of an evolving quantum state. In our work, we have shown that an isolated *d*-dimensional quantum particle with quadratic spectrum exhibits a polynomial complexity growth ${\mathscr{N}}(\tau )={\tau }^{d}$. Uncovering the ${\mathscr{N}}(\tau )$ dependence for realistic situations, accounting for the interactions will establish a mechanism and the corresponding time-scale on which time-reversed states can spontaneously emerge. Another fundamental question is whether it is possible at all to design a quantum algorithm that would perform time-reversal more efficiently than using ${\mathscr{O}}({\mathscr{N}})$ elementary gates. So far, our time-reversal schemes were scrolling one by one through the state components but did not exploit a quantum parallelism in its full power. On a practical side the time-reversal procedure might be helpful for the quantum program testing. Having in hands a multi-qubit quantum computer it is hard to verify that it really has computed the desired result. Indeed, the full tomography of the computed state is an exponentially hard task. Alternatively, making the time-reversal of the anticipated computed state and running the same evolution drives the computer back to its initial state if and only if the computer really made a correct computation. The initial state is typically non-entangled and therefore its verification is an easy task.

## Supplementary information

Supplementary Information for Arrow of time and its reversal on the IBM quantum computer

## Data Availability

All data generated or analyzed during this study are included in this published article and its Supplementary Information file.
