# Supplementary material for: Arrow of time and its reversal on the IBM quantum computer
Source: Sci Rep. 2019 Mar 13;9:4396. doi: 10.1038/s41598-019-40765-6 (PMC6416338; doi:10.1038/s41598-019-40765-6)
Supplement: Supplementary file 1 — Supplementary Information for Arrow of time and its reversal on the IBM quantum computer [file 41598_2019_40765_MOESM1_ESM.pdf]

Supplementary Information for Arrow of time and its reversal on the IBM quantum computer

G. B. Lesovik[1],
I. A. Sadovskyy,[3]
M. V. Suslov,[1]
A. V. Lebedev,[2,3]
V. M. Vinokur[3,*],
[1]Moscow Institute of Physics and Technology,
Institutskii per. 9, Dolgoprudny, 141700, Moscow District, Russia
[2]Theoretische Physik, ETH Zürich
Wolfgang-Pauli-Strasse 27, CH-8093 Zürich, Switzerland
[3]Materials Science Division, Argonne National Laboratory,
9700 S. Cass Avenue, Argonne, Illinois 60637, USA
*To whom correspondence should be addressed; E-mail: vinokour@anl.gov

This PDF file includes:

SI 1. Wave-packet reversal complexity
SI 2. Reversal of the qubit register dynamicsSupplementary
SI 3. Optimal phase shifts arrangement
SI 4. Boolean function time-reversal algorithm
SI 5. Simulation of scattering on a two-level impurity
Tables 1-3
Figure S1

SI 1. Wave-packet reversal complexity

Let a charged particle have one dimensional wave function $\psi(x) \equiv \sqrt{p(x)}e^{i\varphi(x)}$. Consider a fluctuating electromagnetic field potential $V(x,t)$ of the electromagnetic field which is approximated by the N-cell stepwise function $V(x,t) = \sum_{n=1}^{N} I_n(x)V(x_n,t)$, where $I_n(x)$ is an

indicator function of the cell with the index n. Let us assume that during the short time interval a relatively strong non-homogenous fluctuation has emerged and the wave packet $\psi(x)$ acquires the coordinate dependent phase shift $\psi(x) \to \tilde{\psi}(x) = \psi(x)\exp(i\sum_n I_n(x)\phi_n)$, where $\phi_n = \int dt\, eV(x_n,t)/\hbar$. Consider then the specific fluctuation with $\phi_n(x) = -2\varphi(x_n)$ which drives the original wave packet $\psi(x)$ into its approximate complex conjugated form $\tilde{\psi}^*(x)$. The accuracy of such a conjugation procedure is defined through the overlap of the exact conjugated state $\psi^*(x)$ with the approximate conjugated state $\tilde{\psi}^*(x)$, $S = \langle\psi^*(x)|\tilde{\psi}^*(x)\rangle$,

$$S = \sum_{n=1}^{\infty}\int dx\, I_n(x)p(x)\, e^{2i(\varphi(x)-\varphi(x_n))}. \tag{1}$$

Then the probability of the correct reversion is given by $|S|^2$. Assuming that the particle density $p(x)$ changes slowly on the scale of large fluctuations of the particle phase $\varphi(x)$ one arrives at

$$|S|^2 \approx 1 - \frac{1}{3}\sum_{n=1}^{N} p(x_n)\delta x_n \left[\varphi'(x_n)\delta x_n\right]^2, \tag{2}$$

for the sufficiently small δx_n of the cells defined through the condition $g(x_n) \equiv \varphi'(x_n)\delta x_n \ll 1$. Then the error probability ϵ of the incorrect conjugation of the wavepacket is given by $|S|^2 = 1-\epsilon$ and in the continuous limit one has

$$\epsilon = \frac{1}{3}\int dx\, p(x)\, g^2(x). \tag{3}$$

Let us now find the number of cells N needed to approximate the electromagnetic field complex conjugation procedure with a given error probability level ϵ. From the definition $g(x) = \varphi'(x)\delta x$ one has

$$N = \int dx\, \frac{|\varphi'(x)|}{g(x)}. \tag{4}$$

Minimizing the functional $N[g(x)]$ under the constraint Eq. (3) one finds

$$N = \Big(\frac{\lambda^3(\psi)}{3\epsilon}\Big)^{1/2}, \quad \lambda(\psi) \equiv \int dx\, \big(|\psi(x)|^2[\phi'(x)]^2\big)^{1/3}. \tag{5}$$

Generalization of the above result to a d-dimensional case is straightforward

$$N_d = \lambda(\psi)\Big(\frac{\lambda(\psi)}{3\epsilon}\Big)^{d/2}, \quad \lambda(\psi) = \int d^d\vec{x}\, \big(|\psi(\vec{x})|^2\vec{\nabla}^2\varphi(\vec{x})\big)^{d/(d+2)}. \tag{6}$$

Applying these results to the wave packet given by the Eq. (1) of the main text, one obtains $\lambda(\Psi) \sim (\hbar\tau/m)^{2/3}\int dx(f^2(x)x^2)^{1/3}$. We assume that initially at $\tau = 0$ the wave packet has the size L_0, so that $f^2(k) \sim L_0$ for $|k| \leq 1/L_0$ and, therefore, $\lambda(\Psi) \sim (L_\tau/L_0)^{2/3}$ where $L_\tau = \hbar\tau/mL_0$ is the size of the wave packet after the free evolution during the time τ. Therefore, the number of the elementary cells needed to arrange the electromagnetic potential fluctuation which reverses the dynamics of a one dimensional wave packet is linear in τ since $N \sim \epsilon^{-1/2}\, L_\tau/L_0$, see also *(S1)*. For a d-dimensional wave packet the number of cells grows polynomially with τ as $N \sim \epsilon^{-d/2}\,(L_\tau/L_0)^d$.

SI 2. Reversal of the qubit register dynamics

Let the forward time dynamics of the n-qubit register state $|\psi(t)\rangle = \sum_{i=0}^{N-1} \psi_i(t)|i\rangle$ be governed by the Hamiltonian $\hat{H}$, $i\hbar\partial_t|\psi(t)\rangle = \hat{H}|\psi(t)\rangle$. The time-reversal symmetry of the Schrödinger equation implies that if there is a forward time solution $|\psi(t)\rangle$ then the backward time solution $|\tilde{\psi}(t)\rangle$

$$-i\hbar|\tilde{\psi}(t)\rangle = \hat{H}|\tilde{\psi}(t)\rangle \tag{7}$$

also exists and is uniquely defined through the forward time solution via the time-reversal operation $\hat{R}$ such that $|\tilde{\psi}(t)\rangle = \hat{R}|\psi(t)\rangle$. The time-reversal operation $\hat{R}$ is an anti-unitary operation: $\langle\hat{R}\psi_1|\hat{R}\psi_2\rangle = \langle\psi_1|\psi_2\rangle^*$ and can be presented as a product $\hat{R} = \hat{U}_R\hat{K}$ of some unitary operator $\hat{U}_R$ and the complex conjugation operation $\hat{K}$ which we define with respect to the computational basis $|i\rangle$ of the qubit register as

$$\hat{K}\Big(\sum_i \psi_i|i\rangle\Big) = \sum_i \psi_i^*|i\rangle\,. \tag{8}$$

Substituting $|\tilde{\psi}(t)\rangle = \hat{U}_R\hat{K}|\psi(t)\rangle$ into Eq. (7) one finds

$$i\hbar\partial_t|\psi(t)\rangle = \big(\hat{U}_R^\dagger\hat{H}\hat{U}_R\big)^*|\psi(t)\rangle, \tag{9}$$

and therefore the unitary operation $\hat{U}_R$ has to satisfy a relation,

$$\hat{H} = \big(\hat{U}_R^\dagger\hat{H}\hat{U}_R\big)^*. \tag{10}$$

The relation (10) defines the unitary $\hat{U}_R$. Indeed, the hermitian operator $\hat{H}$ can be represented in a form $\hat{H} = \hat{U}_H^\dagger\hat{E}\hat{U}_H$, where $\hat{E}$ is a real diagonal operator and $\hat{U}_H$ is unitary. Then it follows from the Eq. (10)

$$\hat{U}_R = \hat{U}_H^\dagger\hat{U}_H^*. \tag{11}$$

The forward time evolution operator $\hat{U}(\tau) = \exp(-i\hat{H}\tau/\hbar)$ applied to the time reversed state $|\tilde{\psi}(\tau)\rangle$ drives it into the new state

$$\hat{U}(\tau)|\tilde{\psi}(\tau)\rangle = \hat{R}|\psi(0)\rangle. \tag{12}$$

Indeed,

$$\begin{aligned}\hat{U}(\tau)|\tilde{\psi}(\tau)\rangle &\equiv \exp\Big(-\frac{i}{\hbar}\hat{H}\tau\Big)\hat{U}_R\hat{K}\exp\Big(-\frac{i}{\hbar}\hat{H}\tau\Big)|\psi(0)\rangle \\ &= \exp\Big(-\frac{i}{\hbar}\hat{H}\tau\Big)\exp\Big(+\frac{i}{\hbar}\hat{U}_R\hat{H}^*\hat{U}_R^\dagger\tau\Big)\hat{R}|\psi(0)\rangle.\end{aligned} \tag{13}$$

Making use of the explicit form of the $\hat{U}_R$ operator, see Eq. (11), one has $\hat{U}_R\hat{H}^t\hat{U}_R^\dagger = \hat{H}$ that proves Eq. (12). Therefore, in order to restore the original state $|\psi(0)\rangle$ from the time-evolved state $|\psi(\tau)\rangle$ one has to apply the following sequence of operations

$$|\psi(0)\rangle = \hat{R}^{-1}\hat{U}(\tau)\hat{R}\,|\psi(\tau)\rangle\,. \tag{14}$$

SI 3. Optimal phase shifts arrangement

Here we outline an optimal arrangement of the state selective phase shift operations $\hat{\Phi}_i(\varphi) = |i\rangle\langle i|e^{i\varphi}$ entering the complex conjugation operation $\hat{U}_\psi = \prod_{i=0}^{2^n-1} \hat{\Phi}_i(-2\varphi_i)$ for the qubit state $|\psi\rangle = \sum_{i=0}^{2^n-1} |\psi_i|e^{i\varphi_i}|i\rangle$. Let us consider 2^{k-2} operations $\hat{\Phi}_k$ with index k having the same values of two highest bits $b_0 = b_1 = 1$: $k(k') = 2^{n-1} + 2^{n-2} + k'$, $k' = 0, \dots, 2^{n-2} - 1$. Then in the product $\prod_{k'=0}^{2^{n-2}-1} \hat{\Phi}_k(-2\varphi_k)$ one needs to check the values of the bits b_0 and b_1 only once, and this reduces the number of Toffoli gates. This recipe can be recursively repeated for the next lower bits b_2, b_3 and so on, see Fig. 1B. Then the resulting quantum circuit comprises the sequence of nested blocks or subroutines $\mathcal{A}_{11b_2\dots b_{n-1}} \supset \mathcal{A}_{111b_3\dots b_{n-1}} \supset \cdots \supset \mathcal{A}_{1\dots 1b_{n-1}}$ where each subroutine $\mathcal{A}_{1\dots 1b_m\dots b_{n-1}}$ performs the controlled phase shift on all components $|k\rangle$ with first m highest bits equal to 1. As follows from the Fig. 1B, the subroutine $\mathcal{A}_{11\dots b_m\dots b_{n-1}}$ involves two subroutines of the next lower level $\mathcal{A}_{11\dots 1b_{m+1}\dots b_{n-1}}$ and $\mathcal{A}_{11\dots 0b_{m+1}\dots b_{n-1}}$, and two additional Toffoli gates that are needed to check the value of the bit b_{m+1}. Therefore, the number of Toffoli gates $N_{\Lambda_2}\left[\mathcal{A}_{11\dots 1b_m\dots b_{n-1}}\right]$ needed for the implementation of the subroutine $\mathcal{A}_{11\dots 1b_m\dots b_{n-1}}$ obeys the relation $N_{\Lambda_2}\left[\mathcal{A}_{11\dots 1b_m\dots b_{n-1}}\right] = 2 + 2N_{\Lambda_2}\left[\mathcal{A}_{11\dots 1b_{m+1}\dots b_{n-1}}\right]$ with the boundary condition $N_{\Lambda_2}\left[\mathcal{A}_{11\dots 1b_{n-1}}\right] = 2$, that gives $N_{\Lambda_2}\left[\mathcal{A}_{11b_2\dots b_{n-1}}\right] = 2^n - 2$. The full n-qubit complex conjugation procedure $\hat{U}_\psi^{(2)}$ involves four different qubit subroutines $\mathcal{A}_{00b_2\dots b_{n-1}}$, $\mathcal{A}_{01b_2\dots b_{n-1}}$ and so on. This, finally, yields $N_{\Lambda_2}\left[\hat{U}_\psi^{(2)}\right] = 4(2^n - 2)$ and hence $N_\oplus\left[\hat{U}_\psi^{(2)}\right] = 24(2^n - 2) \sim 24\mathcal{N}$.

SI 4. Boolean function time-reversal algorithm

Here we describe the time-reversal procedure of a qubit register based on the arithmetic representation of a n-qubit Boolean function,

$$b_{n-1} \wedge b_{n-2} \wedge \cdots \wedge b_0 = \begin{cases} 1, & b_0 = b_1 = \cdots = b_{n-1} = 1 \\ 0, & \text{overwise} \end{cases} . \tag{15}$$

We find the minimal number of CNOT gates needed for the implementation of this procedure. Let us start with the two-qubit situation where one wishes to reverse the general two-qubit state $|\psi_2\rangle = e^{i\varphi_{00}}|00\rangle + e^{i\varphi_{01}}|01\rangle + e^{i\varphi_{10}}|10\rangle + e^{i\varphi_{11}}|11\rangle$. This requires to implement the complex conjugation procedure, which for a given state can be realized by the two-qubit unitary operation

$$\hat{K}_2 = \sum_{b_0,b_1=0,1} e^{-2i\varphi_{b_1b_0}} |b_1b_0\rangle\langle b_1b_0| \equiv e^{-2i\hat{F}(b_1,b_0)} , \tag{16}$$

where $\hat{F}(b_1, b_0)$ is the two-qubit Boolean function of the form

$$\hat{F}(b_1, b_0) = \varphi_{00}\, \bar{b}_1 \wedge \bar{b}_0 + \varphi_{10}\, b_1 \wedge \bar{b}_0 + \varphi_{01}\, \bar{b}_1 \wedge b_0 + \varphi_{11}\, b_1 \wedge b_0 , \tag{17}$$

and $\bar{b}_i$ denotes the logical negation of the bit b_i, $\bar{b}_i = \mathrm{NOT}(b_i)$. Making use of the arithmetic representation of $b_1 \wedge b_0$, see Eq. (4) of the main text, one finds

$$\hat{F}(b_1, b_0) = \frac{\varphi_{01}+\varphi_{11}}{2} b_0 + \frac{\varphi_{10}+\varphi_{00}}{2} \bar{b}_0 + \frac{\varphi_{10}+\varphi_{11}}{2} b_1 + \frac{\varphi_{01}+\varphi_{10}}{2} \bar{b}_1 \\ - \frac{\varphi_{00}+\varphi_{11}}{2} b_1 \oplus b_0 - \frac{\varphi_{10}+\varphi_{01}}{2} \overline{b_1 \oplus b_0}, \tag{18}$$

where $b_1 \oplus b_0$ denotes a bit summation by modulo 2,

$$b_1 \oplus b_0 = \begin{cases} 0, & b_0 = b_1 \\ 1, & b_0 \neq b_1 \end{cases}. \tag{19}$$

The first four terms in the Eq. (18) correspond to the one-qubit state dependent phase shifts and can be realized only via the single-qubit gates

$$\hat{T}(\alpha) = \begin{pmatrix} 1 & 0 \\ 0 & e^{i\alpha} \end{pmatrix}, \quad \hat{X} = \begin{pmatrix} 0 & 1 \\ 1 & 0 \end{pmatrix}, \tag{20}$$

available on the public IBM quantum computer. The last two-qubit terms in Eq. (18) will require two-qubit CNOT gates. The overall quantum circuit which realizes the unitary operation $\exp\left[-2i\hat{F}(b_1, b_0)\right]$ is described by the following sequence of unitary operations

$$\exp\left[-2i\hat{F}(b_1, b_0)\right] = \mathbf{CNOT}_{0,1} \cdot \Big[\mathbf{TXTX}_1\big(\varphi_{00}+\varphi_{11}, \varphi_{10}+\varphi_{01}\big) \otimes \mathbf{1}_0\Big] \cdot \mathbf{CNOT}_{0,1} \\ \cdot \Big[\mathbf{TXTX}_1\big(-\varphi_{10}-\varphi_{11}, -\varphi_{00}-\varphi_{01}\big) \otimes \mathbf{TXTX}_0\big(-\varphi_{01}-\varphi_{11}, -\varphi_{10}-\varphi_{00}\big)\Big], \tag{21}$$

where $\mathrm{TXTX}_i(\varphi, \bar{\varphi}) \equiv \hat{T}_i(\varphi)\hat{X}_i\hat{T}_i(\bar{\varphi})\hat{X}_i$ is a single-qubit unitary operation which adds specified phase shifts to the state components of the ith qubit: $\mathrm{TXTX}_i(\varphi, \bar{\varphi})\big(a|0_i\rangle + b|1_i\rangle\big) = ae^{i\bar{\varphi}}|0_i\rangle + be^{i\varphi}|1_i\rangle$. The corresponding quantum circuit is shown in the Fig. 1D and involves only two $\mathrm{CNOT}_{0,1}$ gates, where $|b_0\rangle$ qubit serves as control bit and $|b_1\rangle$ as a target.

The above two-qubit complex conjugation procedure can be further extended onto a general n-qubit state. As follows from the Eq. (4) of the main text, the quantum circuit performing complex conjugation of a given n-qubit state requires $\binom{n}{2}$ two-qubit operations,

$$\mathbf{CTXTX}_{i_1 i_2}(\varphi, \bar{\varphi}) \equiv \begin{cases} \hat{T}_{i_2}(\varphi), & b_{i_1} \oplus b_{i_2} = 1 \\ \hat{T}_{i_2}(\bar{\varphi}), & b_{i_1} \oplus b_{i_2} = 0 \end{cases}, \quad 1 \leq i_1 < i_2 \leq n, \tag{22}$$

$\binom{n}{3}$ three-qubit operations,

$$\mathbf{CTXTX}_{i_1 i_2 i_3}(\varphi, \bar{\varphi}) \equiv \begin{cases} \hat{T}_{i_3}(\varphi), & b_{i_1} \oplus b_{i_2} \oplus b_{i_3} = 1 \\ \hat{T}_{i_3}(\bar{\varphi}), & b_{i_1} \oplus b_{i_2} \oplus b_{i_3} = 0 \end{cases}, \quad 1 \leq i_1 < i_2 \leq i_3 \leq n, \tag{23}$$

and so on. The general n-qubit operation $\mathbf{CTXTX}_{i_1 \ldots i_n}(\varphi, \bar{\varphi})$, $1 \leq i_1 < i_2 < \cdots < i_n$ is implemented with the help of $2(n-1)$ CNOT gates as shown on the Fig. S1. Therefore, one

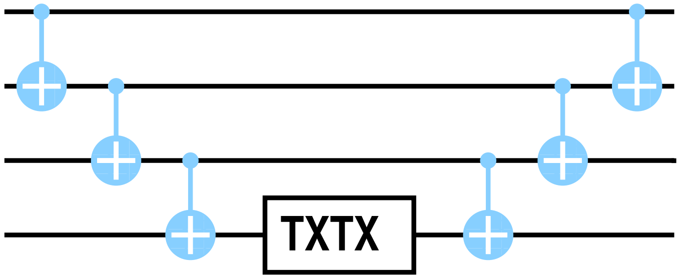

Figure S 1: The quantum circuit which implements the four-qubit quantum gate CTXTX_{0123}. One can check that for any computational basis state $|b_3b_2b_1b_0\rangle$ the state of the elder bit b_3 is given by $b_0 \oplus b_1 \oplus b_2 \oplus b_3$ right after the first ladder CNOT gates. The remaining symmetric half of CNOT gates is required in order to restore the original quantum state of the qubit register.

might conclude that in total $2\sum_{k=2}^{n}(k-1)\binom{n}{k} = 2^n(n-2)+2$ CNOT gates are required in order to implement a n-qubit time-reversal procedure.

However, the number of the CNOT gates can be reduced as far as some of operators $\mathrm{CTXTX}_{i_1i_2\ldots}$ can be grouped together. Consider, for example, the unitary operation $\mathrm{CTXTX}_{12}\cdot\mathrm{CTXTX}_{123}$. Its straightforward implementation requires $4+2$ CNOT gates. A more savvy arrangement is shown in the Fig. 1F of the main text. There the computational state of the second qubit b_1 right after the first $\mathrm{CNOT}_{0,1}$ gate is given by $b_1 \oplus b_2$. This enables one to implement the controlled phase shift CXTXT_{01} right after the first $\mathrm{CNOT}_{0,1}$ operation. At this moment, one need not to restore the original bit values b_0 and b_1 but rather to add the second $\mathrm{CNOT}_{1,2}$, set the third qubit b_3 into the state $b_0 \oplus b_1 \oplus b_2$, and to implement the controlled phase shift CTXTX_{012}. Hence the unitary operation $\mathrm{CTXTX}_{01}\cdot\mathrm{CTXTX}_{012}$ will require the same number of CNOT gates as the operation CTXTX_{012} alone. As a result, the complex conjugation operation of a given 3-qubit state can be implemented using only 8 CNOT gates as shown in Fig. 1F.

The above CNOT optimization technique can be easily generalized to a n-qubit case. Consider a product $\mathrm{CTXTX}_{i_1i_2}\cdot\mathrm{CTXTX}_{i_1i_2i_3}\cdot\mathrm{CTXTX}_{i_1i_2i_3i_4}\cdots\cdot\mathrm{CTXTX}_{i_1i_2i_3i_4\ldots i_n}$ where a sequence of nested strings of the qubit indices $i_1i_2 \subset i_1i_2i_3 \subset \cdots \subset i_1i_2i_3i_4\ldots i_n$ are formed by adding an additional index to the right hand side of a previous string. Then the implementation of this product requires the same number of CNOT gates as the largest $\mathrm{CTXTX}_{i_1i_2i_3i_4\ldots i_n}$ factor of the product. This observation lets us find a number of CNOT gates $N_\oplus\left[\hat{K}_n\right]$ needed to implement the complex conjugation unitary operation $\hat{K}_n$ of a given n-qubit state.

Let us assume that $N_\oplus\left[\hat{K}_{n-1}\right]$ for a $n-1$ qubit register $b_1\ldots b_{n-1}$ is known. Let us add an additional qubit line b_0 and find how many additional operations $\mathrm{CTXTX}(i_1i_2\ldots)$ one needs in order to complete the complex conjugation task for n-qubit register $b_0\ldots b_{n-1}$. Obviously any such additional operation CTXTX_s has its parameter string $s=i_1\ldots i_k$ starting from the index 0, i.e. $i_1=0$. Consider for example $n=4$ case. Then there are seven additional operations,

$$\mathrm{CTXTX}_{0123}\cdot\mathrm{CTXTX}_{012}\cdot\mathrm{CTXT}_{013}\cdot\mathrm{CTXTX}_{02}\cdot\mathrm{CTXTX}_{01}\cdot\mathrm{CTXTX}_{02}\cdot\mathrm{CTXTX}_{03}. \quad (24)$$

Making an optimization procedure one can group these operations as

$$\Big(\mathrm{CTXTX}_{01}\cdot\mathrm{CTXTX}_{012}\cdot\mathrm{CTXTX}_{0123}\Big)\cdot\Big(\mathrm{CTXTX}_{02}\cdot\mathrm{CTXTX}_{023}\Big)\cdot\mathrm{CTXTX}_{013}\cdot\mathrm{CTXTX}_{03}, \quad (25)$$

and hence

$$\begin{aligned} N_\oplus\big[\hat{K}_4\big] = N_\oplus\big[\mathrm{CTXTX}_{0123}\big] + N_\oplus\big[\mathrm{CTXTX}_{023}\big] + N_\oplus\big[\mathrm{CTXTX}_{013}\big] \\ + N_\oplus\big[\mathrm{CTXTX}_{03}\big] + N_\oplus\big[\hat{K}_3\big], \end{aligned} \quad (26)$$

where $N_\oplus\big[\mathrm{CTXTX}_s\big]$ is the number of CNOT gates needed for the operation CTXTX_s. One can note, that only generalized operations CTXTX_s with the inputs strings $s = i_1 \ldots i_k$ where first and last indices are equal to 0 and 3, respectively are counted for the total number of the CNOT gates. Therefore, for a general case, the following relation holds

$$\begin{aligned} N_\oplus\big[\hat{K}_n\big] &= N_\oplus\big[\hat{K}_{n-1}\big] + N_\oplus\big[\mathrm{CTXTX}_{1n}\big] + \sum_{1<k_1<n} N_\oplus\big[\mathrm{CTXTX}_{1k_1n}\big] \quad (27) \\ &+ \sum_{1<k_1<k_2<n} N_\oplus\big[\mathrm{CTXTX}_{1k_1k_2n}\big] + \cdots + N_\oplus\big[\mathrm{CTXTX}_{1\ldots n}\big] \\ &= N_\oplus\big[\hat{K}_{n-1}\big] + \sum_{k=0}^{n-2} 2(k+1)\binom{n-2}{k} = N_\oplus\big[\hat{K}_{n-1}\big] + n2^{n-2}, \quad (28) \end{aligned}$$

and, therefore,

$$N_\oplus\big[\hat{K}_n\big] = (n-1)2^{n-1}, \quad n > 1. \quad (29)$$

SI 5. Simulation of scattering on a two-level impurity

Here we discuss a spinless particle which scatters on a two-level impurity (TLI). The free dynamics of the TLI is governed by a Hamiltonian

$$\hat{H}_\mathrm{i} = \hbar\omega\left(\cos(\alpha)\,\hat{\sigma}_z + \sin(\alpha)\,\hat{\sigma}_x\right). \quad (30)$$

The scattering process is described by the 2×2 scattering matrix $\hat{S}_i$, $i = 0, 1$ whose form depends on the impurity state. The quantum state of the particle-impurity system can be described as the two-bit state $|\psi\rangle = \sum_{b_0,b_1=0,1} A_{b_1b_0}|b_1\rangle \otimes |b_0\rangle$ where the first qubit describes the TLI and the second one describes the propagation direction of an incoming/scattered particle. Let the system start in the state $|\psi(0)\rangle = |0\rangle \otimes |L\rangle$ with the particle coming from the left. Let after the time $\tau > 0$ the particle be scattered on the TLI. The resulting state $|\psi(\tau)\rangle$ is generated by the sequence of unitary operations $|\psi(\tau)\rangle = \hat{S}_\psi \cdot \big[\hat{U}_\mathrm{i}(\tau) \otimes \mathbf{1}\big]\,|\psi(0)\rangle$, where the unitary operator $\hat{U}_\mathrm{i}(\tau) \equiv \exp\big(-i\hat{H}_\mathrm{i}\tau/\hbar\big)$ describes the free evolution of TLI and

$$\hat{S}_\psi = |0\rangle\langle 0| \otimes \hat{S}_0 + |1\rangle\langle 1| \otimes \hat{S}_1 \quad (31)$$

describes the state dependent scattering process of the incoming particle. The unitary operator $\hat{U}_{\rm i}(\tau) = \exp\big[-i\omega\tau(\hat{\sigma}_x\cos\alpha + \hat{\sigma}_z\sin\alpha)\big]$ is symmetric. In the absence of the magnetic field, the scattering operator $\hat{S}_\psi$ is symmetric as well. Let the state freely evolve after the scattering at the $t = \tau$ during the same time period τ. Then the resulting state $|\psi(2\tau)\rangle = [\hat{U}_{\rm i}(\tau)\otimes\mathbf{1}]|\psi(\tau)\rangle$ can be generated from the initial state $|\psi(0)\rangle$ by the *symmetric* 2-qubit unitary operator

$$\hat{U}_{\rm 2bit} = \big[\hat{U}_{\rm i}(\tau)\otimes\mathbf{1}\big]\cdot\hat{S}_\psi\cdot\big[\hat{U}_{\rm i}(\tau)\otimes\mathbf{1}\big]. \tag{32}$$

Therefore, as we have already discussed in SI 2, the time reversal procedure of the 2-qubit state $|\psi(2\tau)\rangle$ requires only the unitary implementation of the complex conjugation operation $|\psi(2\tau)\rangle \to |\psi^*(2\tau)\rangle$.

Our goal is to implement the unitary operation $\hat{U}_{\rm 2bit}$ via the set of quantum gates available on the IBM public quantum computer. The only available two-qubit gate is the CNOT_{b_c,b_t} gate, where b_c the qubit serves as a control and b_t qubit serves a target. Among the standard 1-qubit gates we will need two available generalized 1-qubit gates: the relative phase shift gate $\hat{T}(\alpha)$, introduced in the SI 4 and the full 1-qubit unitary rotation

$$\hat{U}_3(\theta,\alpha,\beta) \equiv \hat{T}(\alpha)\cdot\hat{R}(\theta)\cdot\hat{T}(\beta), \tag{33}$$

where

$$\hat{R}(\theta) = \begin{pmatrix} \cos\frac{\theta}{2} & -\sin\frac{\theta}{2} \\ \sin\frac{\theta}{2} & \cos\frac{\theta}{2} \end{pmatrix}. \tag{34}$$

Any 2×2 unitary matrix $\hat{U}$ can be represented in the form (33) up to some phase factor: $\hat{U} = e^{i\delta}\hat{U}_3(\theta,\alpha,\beta)$. In particular, any symmetric 2×2 unitary matrix $\hat{U} = \hat{U}^t$ has the form $e^{i\delta}\hat{U}_3(\theta,\alpha,\alpha{+}\pi)$. Therefore, a given set of matrices $\hat{U}_{\rm i}(\tau)$, $\hat{S}_0$ and $\hat{S}_1$ entering into the definition of the model can be presented as

$$\hat{U}_{\rm i}(\tau) = e^{i\delta}\hat{U}_3(\xi,\eta,\eta+\pi), \tag{35}$$

$$\hat{S}_i = e^{i\delta_i}\hat{U}_3(\theta_i,\varphi_i,\varphi_i+\pi), \quad i = 0,1. \tag{36}$$

The phase exponent $e^{i\phi}$ gives only a trivial common phase factor for the system state and will be omitted in what follows. Without any loss of generality we assume $\delta_0 = 0$ as well.

Next, let us construct the 2-qubit operation $\hat{S}_\psi$ using as less CNOT gates as possible. It turns out that $\hat{S}_\psi$ can be constructed with the help of only two CNOT gates. Indeed,

$$\hat{S}_\psi = \Big(|1\rangle\langle 1|\otimes\hat{S}_1\hat{S}_0^\dagger + |0\rangle\langle 0|\otimes\mathbf{1}\Big)\cdot\big[\mathbf{1}\otimes\hat{S}_0\big] \equiv \Lambda_{b_1,b_0}(\hat{S}_1\hat{S}_0^\dagger)\cdot\big[\mathbf{1}\otimes\hat{S}_0\big], \tag{37}$$

where $\Lambda_{b_1,b_0}(\hat{W})$ is a controlled $\hat{W}$-gate,

$$\Lambda_{b_c,b_t}(\hat{W})\big(|b_c\rangle\otimes|b_t\rangle\big) = \begin{cases} |b_c\rangle\otimes\hat{W}|b_t\rangle & ,b_1 = 1 \\ |b_c\rangle\otimes|b_t\rangle & ,b_1 = 0 \end{cases}. \tag{38}$$

The unitary matrix $\hat{W} = \hat{S}_1 \hat{S}_0^\dagger \equiv e^{i\delta} \hat{U}_3(\theta, \alpha, \beta)$ can be represented as,

$$\hat{W} = e^{i(\delta + \frac{\alpha+\beta}{2})} \hat{T}(\alpha) \hat{R}(\frac{\theta}{2})\, \hat{\sigma}_x\, \hat{R}(-\frac{\theta}{2}) \hat{T}(-\frac{\alpha+\beta}{2})\, \hat{\sigma}_x\, \hat{T}(\frac{\beta-\alpha}{2}). \tag{39}$$

The advantage of the latter representation is that if one replaces in the Eq. (39) two Pauli matrices $\hat{\sigma}_x$ by the identity operator, one gets a phase shift $\exp[i(\delta + \frac{\alpha+\beta}{2})]$ only. Therefore,

$$\begin{aligned} \Lambda_{b_1,b_0}(\hat{W}) = & \big[\hat{T}(\delta + \frac{\alpha+\beta}{2}) \otimes \hat{U}_3(\frac{\theta}{2}, \alpha, 0)\big] \\ & \cdot \mathrm{CNOT}_{b_1,b_0} \cdot \big[\mathbf{1} \otimes \hat{U}_3(-\frac{\theta}{2}, 0, -\frac{\alpha+\beta}{2})\big] \cdot \mathrm{CNOT}_{b_1,b_0} \cdot \big[\mathbf{1} \otimes \hat{T}(\frac{\beta-\alpha}{2})\big], \end{aligned} \tag{40}$$

and the whole evolution operator, see Eq. (32) can be presented as,

$$\hat{U}_{\text{2bit}} = \big[\hat{U}_3(\xi, \eta, \eta+\pi) \otimes \mathbf{1}\big] \cdot \Lambda_{b_1,b_0}(\hat{S}_1 \hat{S}_0^\dagger) \cdot \big[\hat{U}_3(\xi, \eta, \eta+\pi) \otimes \hat{U}_3(\theta_0, \varphi_0, \varphi_0 + \pi)\big]. \tag{41}$$

The corresponding 2-qubit quantum circuit is shown on a Fig. 1C.

Similarly, we consider a 3-qubit model describing the scattering of two particles on a TLI. We assume that particles arrive to the TLI with the time separation τ,

$$\begin{aligned} \hat{U}_{\text{3bit}} = & \big[\hat{U}_{\text{i}}(\tau) \otimes \mathbf{1} \otimes \mathbf{1}\big] \cdot \big[|0\rangle\langle 0| \otimes \mathbf{1} \otimes \hat{S}_0 + |1\rangle\langle 1| \otimes \mathbf{1} \otimes \hat{S}_1\big] \\ & \cdot \big[\hat{U}_{\text{i}}(\tau) \otimes \mathbf{1} \otimes \mathbf{1}\big] \cdot \big[|0\rangle\langle 0| \otimes \hat{S}_0 \otimes \mathbf{1} + |1\rangle\langle 1| \otimes \hat{S}_1 \otimes \mathbf{1}\big] \cdot \big[\hat{U}_{\text{i}}(\tau) \otimes \mathbf{1} \otimes \mathbf{1}\big], \end{aligned} \tag{42}$$

where the first (eldest) bit describes the state of the TLI and the second and third qubits describe the scattering state of the first and second particles correspondingly. The quantum circuit which implements the evolution operator $\hat{U}_{\text{3bit}}$ is shown in the Fig. 1E of the main text.

SI 6. Time-reversal experiment

In the simulation experiment we choose fixed scattering matrices of the two-level impurity (TLI),

$$\hat{S}_0 = \begin{bmatrix} \frac{1}{2} & \frac{\sqrt{3}}{2} \\ \frac{\sqrt{3}}{2} & -\frac{1}{2} \end{bmatrix}, \quad \hat{S}_1 = \begin{bmatrix} \frac{\sqrt{3}}{2} & \frac{1}{2}e^{i\pi/3} \\ \frac{1}{2}e^{i\pi/3} & -\frac{\sqrt{3}}{2}e^{2\pi i/3} \end{bmatrix}, \tag{43}$$

for the $|0\rangle$ and $|1\rangle$ impurity states correspondingly. Then the state dependent scattering operator $\hat{S}_\psi$, see Eqs. (37) and (40), is given by the following sequence of quantum gates,

$$\begin{aligned} \hat{S}_\psi \approx & \big[\mathbf{1} \otimes \hat{U}_3(0.723, -1.27)\big] \cdot \mathrm{CNOT}_{1,2} \cdot \big[\hat{T}(1.047) \otimes \hat{U}_3(-0.723, 0, -0.523)\big] \\ & \cdot \mathrm{CNOT}_{1,2}\big[\mathbf{1} \otimes \hat{T}(1.761)\big] \cdot \big[\mathbf{1} \otimes \hat{U}_3(\frac{2\pi}{3}, 0, \pi)\big], \end{aligned} \tag{44}$$

where the first (control) qubit describes a state of TLI and the second (target) qubit describes a scattering state of the particle, $\hat{U}_3(\alpha, \varphi, \lambda)$ and $\hat{T}(\varphi)$ are generalized one-qubit gates available on the IBM quantum computer.

The free evolution operator $\hat{U}_{\rm i}(\tau) = \exp(-i\hat{H}_{\rm i}\tau/\hbar)$ with $\hat{H}_{\rm i} = \hbar\omega(\cos\alpha\,\hat{\sigma}_z + \sin\alpha\hat{\sigma}_x)$ is parameterized by two parameters $\omega\tau$ and α. The unitary operator $\hat{U}_{\rm i}(\tau)$ is symmetric and for a fixed values of $\omega\tau$ and α can be presented in the form,

$$\hat{U}_{\rm i}(\tau) = e^{i\delta}\hat{U}_3(\xi, \eta, \eta + \pi), \qquad \xi = \xi(\omega\tau, \alpha), \eta = \eta(\omega\tau, \alpha), \tag{45}$$

where $e^{i\delta}$ some phase factor which changes only an overall phase of the qubit register; ξ and η are parameters which uniquely defined by $\omega\tau$ and α. In the following we choose $\omega\tau = \pi/6$ and vary the parameter α among four values $\pi/6$, $\pi/4$, $\pi/3$ and $\pi/2$ with the corresponding gate parameters,

$$\begin{aligned} &\xi\left(\frac{\pi}{6}, \frac{\pi}{6}\right) \approx 0.505, \quad \eta\left(\frac{\pi}{6}, \frac{\pi}{6}\right) \approx -1.107, \\ &\xi\left(\frac{\pi}{6}, \frac{\pi}{4}\right) \approx 0.723, \quad \eta\left(\frac{\pi}{6}, \frac{\pi}{4}\right) \approx -1.183, \\ &\xi\left(\frac{\pi}{6}, \frac{\pi}{3}\right) \approx 0.896, \quad \eta\left(\frac{\pi}{6}, \frac{\pi}{3}\right) \approx -1.290, \\ &\xi\left(\frac{\pi}{6}, \frac{\pi}{2}\right) \approx 1.047, \quad \eta\left(\frac{\pi}{6}, \frac{\pi}{2}\right) \approx -\pi/2. \end{aligned} \tag{46}$$

The occurrence rates of the computational basis states for 2-qubit and 3-qubit experiments are shown in Tables 1 and 2 for the different input parameters of the model. The 2-qubit experiment used q_1 and q_2 qubit lines of the 'ibmqx4' five qubit quantum processor. The 3-qubit experiment used in addition a q_0 qubit line. In both experiments the q_2 qubit line has modeled a state of TLI. The calibration state of the quantum computer was the same for all experiments. The qubit's relaxation times T_1, coherence times T_2, readout errors ϵ_r and one-qubit gate errors ϵ_1 for each qubit line are shown in the Table. 3. The errors of the CNOT gates $\mathrm{CNOT}_{q2,q0}$, $\mathrm{CNOT}_{q2,q1}$ and $\mathrm{CNOT}_{q1,q0}$ used in the experiments are $\epsilon_{g20} = 1.91\%$, $\epsilon_{g21} = 2.68\%$ and $\epsilon_{g10} = 1.70\%$ respectively. These processor's state parameters allows us to estimate a theoretical value of a time-reversal fidelity $F = |\langle 0 \ldots 0|\tilde{\psi}_0\rangle|^2$, where $|\tilde{\psi}_0\rangle$ is a final state of the qubit register. For the used gate arrangement one has,

$$F_{\rm 2bit}^{\rm theor} = (1-\epsilon_{g21})^6(1-\epsilon_{r1})(1-\epsilon_{r2}) \approx 79.6\% \tag{47}$$

$$F_{\rm 3bit}^{\rm theor} = (1-\epsilon_{g21})^6(1-\epsilon_{g20})^6(1-\epsilon_{g10})^4(1-\epsilon_{r0})(1-\epsilon_{r1})(1-\epsilon_{r2}) \approx 63.4\%, \tag{48}$$

while the experimentally observed values of the time-reversal fidelity are shown in Tables. 1 and 2.

Table 1:

$\omega\tau$	α	$\|00\rangle$	$\|10\rangle$	$\|01\rangle$	$\|11\rangle$	F
$\pi/6$	$\pi/6$	6949	437	562	244	$84.8 \pm 0.4\%$
$\pi/6$	$\pi/4$	6916	440	576	260	$84.4 \pm 0.4\%$
$\pi/6$	$\pi/3$	6983	370	560	279	$85.2 \pm 0.4\%$
$\pi/6$	$\pi/2$	6950	338	551	353	$84.8 \pm 0.4\%$

Table 2:

$\omega\tau$	α	$\|000\rangle$	$\|001\rangle$	$\|010\rangle$	$\|011\rangle$	$\|100\rangle$	$\|101\rangle$	$\|110\rangle$	$\|111\rangle$	F
$\pi/6$	$\pi/6$	3909	1380	1069	487	482	309	332	224	$47.7 \pm 0.5\%$
$\pi/6$	$\pi/4$	3934	1157	981	380	618	360	407	355	$48.0 \pm 0.5\%$
$\pi/6$	$\pi/3$	3957	832	884	327	859	359	531	443	$48.3 \pm 0.5\%$
$\pi/6$	$\pi/2$	3879	355	1050	425	964	418	630	471	$47.3 \pm 0.5\%$

Supplementary references

S1 Lesovik, G. B. On the law of increasing entropy and the cause of the dynamics irreversibility of quantum systems. *JETP Lett.* **98**, 184–189 (2013).

Table 3:

q_n	$T_1(\mu\text{s})$	$T_2(\mu\text{s})$	$\epsilon_r(\%)$	$\epsilon_1(\%)$
q_0	52.4	47.3	4.2	0.077
q_1	58.0	40.6	3.6	0.103
q_2	46.9	47.4	2.8	0.137
